# Risk of New‐Onset Ischaemic and Haemorrhagic Stroke in Patients With Type 2 Diabetes With Chronic Kidney Disease on SGLT‐2 Inhibitor Users: A Population‐Based Cohort Study

**DOI:** 10.1002/dmrr.70122

**Published:** 2026-01-14

**Authors:** Ya‐Hui Lin, Tsung‐Kun Lin, Pei‐Lun Liao, Tsung‐Yuan Yang, Gwo‐Ping Jong

**Affiliations:** ^1^ College of Nursing Central Taiwan University of Science and Technology Taichung Taiwan (ROC); ^2^ Department of Pharmacy Taichung Armed Forces General Hospital Taichung Taiwan (ROC); ^3^ Department of Pharmacy Tre‐Service General Hospital Taipei Taiwan (ROC); ^4^ School of Pharmacy National Defense Medical University Taipei Taiwan (ROC); ^5^ Department of Medical Research Chung Shan Medical University Hospital Taichung Taiwan (ROC); ^6^ Department of Internal Medicine Chung Shan Medical University Hospital Taichung Taiwan (ROC); ^7^ Institute of Medicine Chung Shan Medical University Taichung Taiwan (ROC)

**Keywords:** haemorrhagic stroke, ischaemic stroke, SGLT2 inhibitors, type 2 diabetes

## Abstract

**Background:**

Type 2 diabetes (T2D) and chronic kidney disease (CKD) increase the risk of ischaemic and haemorrhagic strokes. However, the effect of sodium‐glucose cotransporter 2 inhibitors (SGLT2i) on reducing the risk of ischaemic and haemorrhagic strokes in patients with T2D and CKD remains unclear. Thus, this study was conducted to explore the role of SGLT2i in the prevention of ischaemic and haemorrhagic strokes.

**Methods:**

In this retrospective cohort study, Cox regression analysis was employed to examine the hazard ratio (HR) between users and nonusers of SGLT2i on incident ischaemic and haemorrhagic strokes following 1:1 propensity score matching. The Kaplan–Meier method was used to determine the risk of study outcome over time between users and nonusers of SGLT2i. Finally, a sensitivity analysis of the HR was performed between users and nonusers of SGLT2i on incident ischaemic and haemorrhagic strokes after 1:2 sex and age matching.

**Results:**

After 1:1 propensity score matching of patients by age, sex, T2D duration, and comorbidities, 107,819 users of SGLT2i and 107,819 nonusers were enrolled for analysis. SGLT2i therapy was associated with significantly reduced incidence of ischaemic and haemorrhagic strokes (HR 0.86, [95% CI, 0.81–0.90]; HR 0.80, [95% CI, 0.74–0.87]). Furthermore, the HR was even more significant in the sensitivity test for incident ischaemic and haemorrhagic strokes.

**Conclusion:**

SGLT2i reduced the risk of incident ischaemic and haemorrhagic strokes among patients with T2D and CKD. The protective profile of the SGLT2i against incident ischaemic and haemorrhagic strokes makes it a clinical option for those with T2D with CKD.

## 1 Introduction

Over the past 2 decades, the global incidence and prevalence of stroke have increased, resulting in a significant health burden worldwide [1, 2]. Epidemiological studies have shown that type 2 diabetes (T2D) and chronic kidney disease (CKD) are at an increased risk of stroke, including all its subtypes [3, 4]. Adults with T2D with CKD are more likely to experience a stroke at a younger age, with worse outcomes and higher risk for recurrence than those without T2D but with CKD [5, 6]. Patients with T2D who also have CKD and experience either incident ischaemic or haemorrhagic stroke have a higher risk of mortality than those with T2D alone [7, 8]. Thus, ischaemic or haemorrhagic stroke prevention should be a major concern in the clinical management of patients with T2D and CKD.

Sodium‐glucose cotransporter 2 inhibitors (SGLT2i) lower blood glucose levels in patients with T2D and CKD and reduce mortality as well as the risk of cardiovascular diseases, primarily by reducing the risk of hospitalisation for heart failure and improving kidney outcomes [9, 10]. However, the Canagliflozin and Renal Events in Diabetes With Established Nephropathy Clinical Evaluation (CREDENCE) Study Programme demonstrated the neutral risk of ischaemic and haemorrhagic strokes in patients with T2D treated with canagliflozin [11]. Conversely, some meta‐analyses or systematic reviews have demonstrated a significant decrease in the risk of haemorrhagic stroke in patients with T2D treated with SGLT2i; however, they did not demonstrate ischaemic stroke [12, 13, 14]. To date, the role of these SGLT2i in ischaemic and haemorrhagic stroke prevention in patients with T2D and CKD remains unclear. Therefore, a retrospective study was conducted to evaluate the risk of ischaemic and haemorrhagic strokes associated with the use of SGLT2i in patients with T2D and CKD using real‐world data from the Taiwan National Health Insurance database.

## 2 Methods

### 2.1 Study Population

We used claims data of Taiwan Bureau of National Health Insurance (BNHI) from the Health and Welfare Data Science Centre between 2014 and 2019. The Taiwan BNHI covers more than 99% of the Taiwan population's residents and provides comprehensive medical care [15]. These data include patient identification such as numbers, sex, age, three diagnostic codes, medical expenditures, hospital, and prescriptions such as the quantity and expenditure for all drugs, drug dose, operations, and treatments.

This study was approved by the Institutional Review Board of the Chung Shan Medical University Hospital (CS1‐21037). The requirement for informed consent was waived because the database was anonymised and de‐identified by the Ethics Committee for this study. This study was conducted in accordance with the Reporting of Observational Studies in Epidemiology reporting guidelines.

### 2.2 Study Design

**FIGURE 1:**
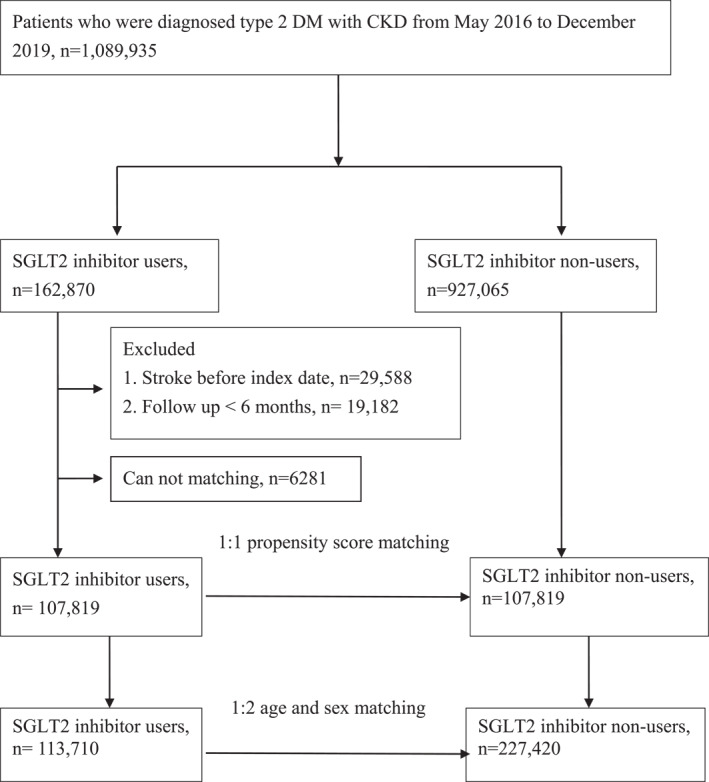
Patient flow chart.

This study enrolled patients aged 20 and older with T2D and CKD who were newly prescribed SGLT2i during the index date period from the Taiwan BNHI between May, 2016 and December 2019. T2D and CKD were identified using the International Classification of Diseases, 10th Revision, Clinical Modification (ICD‐10‐CM) codes E11.22. The participants had to meet at least one of the following criteria: (1) had two or more outpatient visits within 6 months with a diagnosis of T2D and CKD, (2) continuously received antidiabetic medication for more than 6 months during the study period, or (3) had one or more inpatient admissions with a diagnosis of T2D with CKD. Comorbidities related to ischaemic (ICD‐10‐CM code I63) and haemorrhagic stroke (ICD‐10‐CM code I61–I62)were recorded and included coronary heart disease (ICD‐10‐CM code I20–I25), hypertension (ICD‐10‐CM code I10), cancer (ICD‐10‐CM code C00‐C97), atrial fibrillation and flutter (ICD‐10‐CM code I48), chronic obstructive pulmonary disease (ICD‐10‐CM code J44), hyperlipidaemia (ICD‐9‐CM code E78.1–E78.5), chronic liver disease (ICD‐10‐CM code K71, K75, K76), and rheumatoid arthritis (ICD‐9‐CM code M05). Exclusion criteria included (1) a prior history of stroke before index date and (2) a period of follow‐up less than 6 months. The index date was defined as the first SGLT2i prescription during the study period for the SGLT2i group and the index date for controls was their matched case's index date. During the index period, a total of 162,870 patients with T2D with CKD were newly prescribed SGLT2i. After 1:1 propensity score matching, the patients were divided into 107,819 SGLT2i users and 107,819 SGLT2i non‐users to compare the outcomes. A sensitivity analysis using age and sex at a ratio of 1:2 matching was also performed. A flowchart of the study cohort is shown in Figure 1.

### 2.3 Study Outcomes

We obtained information on baseline covariates, including age, sex, comorbidities, and concurrent medication through Taiwan BNHI data. The comorbidity was determined by diagnostic codes first time appearing three months before the index date, and medication use was assessed during a 90‐day baseline period. Furthermore, the study endpoint was the development of ischaemic or haemorrhagic stroke, defined as the first occurrence of an ischaemic or haemorrhagic stroke code in inpatient or outpatient claim records during follow‐up. All patients were followed up from the index date to the event occurrence date, participant death, or the end of the follow‐up period (31 December 2019), whichever occurred first.

### 2.4 Statistical Analysis

The baseline characteristics of the two treatment groups are presented as mean ± standard deviation for continuous variables and compared using Student's *t*‐test. Categorical variables are presented as numbers and percentages and were compared using the chi‐square test. After the propensity score matching analysis, an absolute standardised difference (ASD) of < 0.1 between the two groups was considered to indicate negligible differences for each covariate. The hazard ratios (HRs) of the SGLT2i group compared with non‐SGLT2i users for the endpoints were calculated using Cox proportional hazards regression analysis. The cumulative incidence of endpoints was plotted using the Kaplan–Meier method, and the log‐rank test was used to compare the two groups. Furthermore, a sensitivity analysis using age and sex at a ratio of 1:2 matching was also conducted to test the robustness of our primary findings. Statistical significance was set at *p* < 0.05. All analyses were performed using SAS software (version 9.3; SAS Institute Inc., Cary, NC, USA).

## 3 Results

### 3.1 Baseline Characteristics

In total, 162,870 individuals were eligible for the study after 1:1 propensity score matching, with 107,819 in the SGLT2i group and 107,819 in the non‐SGLT2i group. The baseline characteristics were well balanced on sex, age and duration of T2D, with an ASD of < 0.1 between the two groups (Table 1). Patients in the SGLT2i group had a higher percentage of coronary artery disease, liver disease, cancer, chronic obstructive pulmonary disease, atrial fibrillation and flutter, and rheumatoid arthritis than those in the non‐SGLT2i group. There were also more concurrent medications used except for statins, sulfonylureas, dipeptidyl peptidase‐4 inhibitors, and glucagon‐like peptide‐1 agonists (Table 1).

**TABLE 1:** Baseline characteristics of all patients.

	1:1 propensity score matching			2:1 sex and age matching		
	Non‐SGLT2i	SGLT2i	ASD	Non‐SGLT2i	SGLT2i	ASD
*N*	107,819	107,819		227,420	113,710	
Sex			0.0037			0.0000
Female	47,020 (43.61%)	47,218 (43.79%)		99,414 (43.71%)	49,707 (43.71%)	
Male	60,799 (56.39%)	60,601 (56.21%)		128,006 (56.29%)	64,003 (56.29%)	
Age			0.0542			0.0000
< 50	23,540 (21.83%)	23,030 (21.36%)		48,582 (21.36%)	24,291 (21.36%)	
50–59	29,681 (27.53%)	29,550 (27.41%)		62,280 (27.39%)	31,140 (27.39%)	
60–69	34,610 (32.10%)	34,858 (32.33%)		73,498 (32.32%)	36,749 (32.32%)	
≥ 70	19,988 (18.54%)	20,381 (18.90%)		43,060 (18.93%)	21,530 (18.93%)	
Mean ± SD	58.44 ± 11.89	58.29 ± 12.23				0.0000
Comorbidities						
Hypertension	63,109 (58.53%)	62,749 (58.20%)	0.0068	123,747 (54.41%)	66,644 (58.61%)	0.0847
CAD	16,263 (15.08%)	16,506 (15.31%)	0.0063	27,388 (12.04%)	18,224 (16.03%)	0.1149
Hyperlipidaemia	69,674 (64.62%)	68,957 (63.96%)	0.0139	126,752 (55.73%)	73,488 (64.63%)	0.1824
Liver disease	11,618 (10.78%)	12,006 (11.14%)	0.0115	24,760 (10.89%)	12,646 (11.12%)	0.0075
Cancer	5051 (4.68%)	5351 (4.96%)	0.0130	13,905 (6.11%)	5539 (4.87%)	0.0546
COPD	3977 (3.69%)	4266 (3.96%)	0.0140	8994 (3.95%)	4536 (3.99%)	0.0018
Atrial fibrillation and flutter	1477 (1.37%)	1569 (1.46%)	0.0072	2662 (1.17%)	1713 (1.51%)	0.0292
Rheumatoid arthritis	601 (0.56%)	690 (0.64%)	0.0107	1810 (0.80%)	707 (0.62%)	0.0208
Medication						
NSAIDs	60,782 (56.37%)	61,336 (56.89%)	0.0104	125,976 (55.39%)	65,002 (57.16%)	0.0357
Corticosteroids	19,610 (18.19%)	20,311 (18.84%)	0.0167	42,291 (18.60%)	21,507 (18.91%)	0.0081
PPI	7010 (6.50%)	7427 (6.89%)	0.0155	16,549 (7.28%)	7862 (6.91%)	0.0141
H2 receptor	27,335 (25.35%)	28,033 (26.00%)	0.0148	59,125 (26.00%)	29,637 (26.06%)	0.0015
Aspirin	27,879 (25.86%)	28,024 (25.99%)	0.0031	48,742 (21.43%)	30,409 (26.74%)	0.1244
Statins	74,605 (69.19%)	73,860 (68.50%)	0.0149	127,254 (55.96%)	79,156 (69.61%)	0.2854
Biguanides	62,122 (57.62%)	63,579 (58.97%)	0.0274	104,614 (46.00%)	68,806 (60.51%)	0.2939
Sulfonylureas	43,888 (40.71%)	43,225 (40.09%)	0.0125	71,427 (31.41%)	47,281 (41.58%)	0.2125
Alpha glucosidase inhibitors	17,827 (16.53%)	18,342 (17.01%)	0.0128	24,399 (10.73%)	21,092 (18.55%)	0.2226
Thiazolidinediones	17,402 (16.14%)	17,640 (16.36%)	0.0060	21,121 (9.29%)	20,865 (18.35%)	0.2649
DPP4 inhibitors	42,268 (39.20%)	40,860 (37.90%)	0.0268	59,662 (26.23%)	45,203 (39.75%)	0.2905
Insulin	26,864 (24.92%)	27,127 (25.16%)	0.0056	44,712 (19.66%)	29,816 (26.22%)	0.1565
GLP‐1 agonists	2590 (2.40%)	2493 (2.31%)	0.0059	3823 (1.68%)	2752 (2.42%)	0.0522

Abbreviations: ASD, absolute standardised difference; CAD, Coronary Artery Disease; COPD, chronic obstructive pulmonary disease; DPP4, Dipeptidyl peptidase‐4; GLP‐1, Glucagon‐like peptide‐1; NSAIDs, non‐steroidal anti‐inflammatory drugs; PPI, proton‐pump inhibitor.

### 3.2 The Relative Risk of Incident Ischaemic and Haemorrhagic Stroke in a Propensity Score Matching

The median follow‐up period was 2.1 years in the SGLT2i group and 2.0 years in the non‐SGLT2i group. The incidence rates of ischaemic and haemorrhagic stroke were 9.25 per 10,000 person‐months (95% CI 8.89–9.63) and 4.15 per 10,000 person‐months (95% CI 3.91–4.41) respectively for SGLT2i users compared with 10.83 (95% CI 10.43–11.24) and 5.17 per 10,000 person‐months (95% CI 4.90–5.46), respectively, for non‐SGLT2i users. There was a significantly lower incidence rate of ischaemic and haemorrhagic stroke in the SGLT2i group compared with the non‐SGLT2i group ([crude HR: 0.86; 95% CI: 0.81–0.90]; [crude HR: 0.80; 95% CI: 0.74–0.87], respectively; Tables 2 and 3). The results were consistent after adjustments for sex, age, the index year, comorbidities, and concurrent medication at baseline ([adjusted HR: 0.84; 95% CI: 0.80–0.89]; [adjusted HR: 0.80; 95% CI: 0.73–0.86]).

**TABLE 2:** Incidence rate of ischaemic stroke.

	1:1 propensity score matching		2:1 sex and age matching	
	Non‐SGLT2i	SGLT2i	Non‐SGLT2i	SGLT2i
*N*	107,819	107,819	227,420	113,710
Follow up person months	2,519,882	2,542,061	5,243,195	2,685,779
New case	2729	2352	5873	2498
Incidence rate^a^ (95% C.I.)	10.83 (10.43–11.24)	9.25 (8.89–9.63)	11.20 (10.92–11.49)	9.30 (8.94–9.67)
Crude relative risk (95% C.I.)	Reference	0.86 (0.81–0.90)	Reference	0.83 (0.79–0.87)
Adjusted HR^b^ (95% C.I.)	Reference	0.84 (0.80–0.89)	Reference	0.85 (0.81–0.89)

^a^ Incidence rate, per 10,000 person‐months.

^b^ Adjusted hazard ratio, the covariates including sex, age, year of index, co‐morbidities, and medication at baseline.

**TABLE 3:** Incidence rate of haemorrhage stroke.

	1:1 propensity score matching		2:1 sex and age matching	
	Non‐SGLT2i	SGLT2i	Non‐SGLT2i	SGLT2i
*N*	107,819	107,819	227,420	113,710
Follow up person months	2,541,593	2,560,197	5,286,984	2,705,132
New case	1314	1062	2979	1131
Incidence rate^a^ (95% C.I.)	5.17 (4.90–5.46)	4.15 (3.91–4.41)	5.63 (5.44–5.84)	4.18 (3.94–4.43)
Crude relative risk (95% C.I.)	Reference	0.80(0.74–0.87)	Reference	0.74(0.69–0.80)
Adjusted HR^b^ (95% C.I.)	Reference	0.80(0.73–0.86)	Reference	0.80(0.74–0.86)

^a^ Incidence rate, per 10,000 person‐months.

^b^ Adjusted hazard ratio, the covariates including sex, age, year of index, co‐morbidities, and medication at baseline.

**FIGURE 2:**
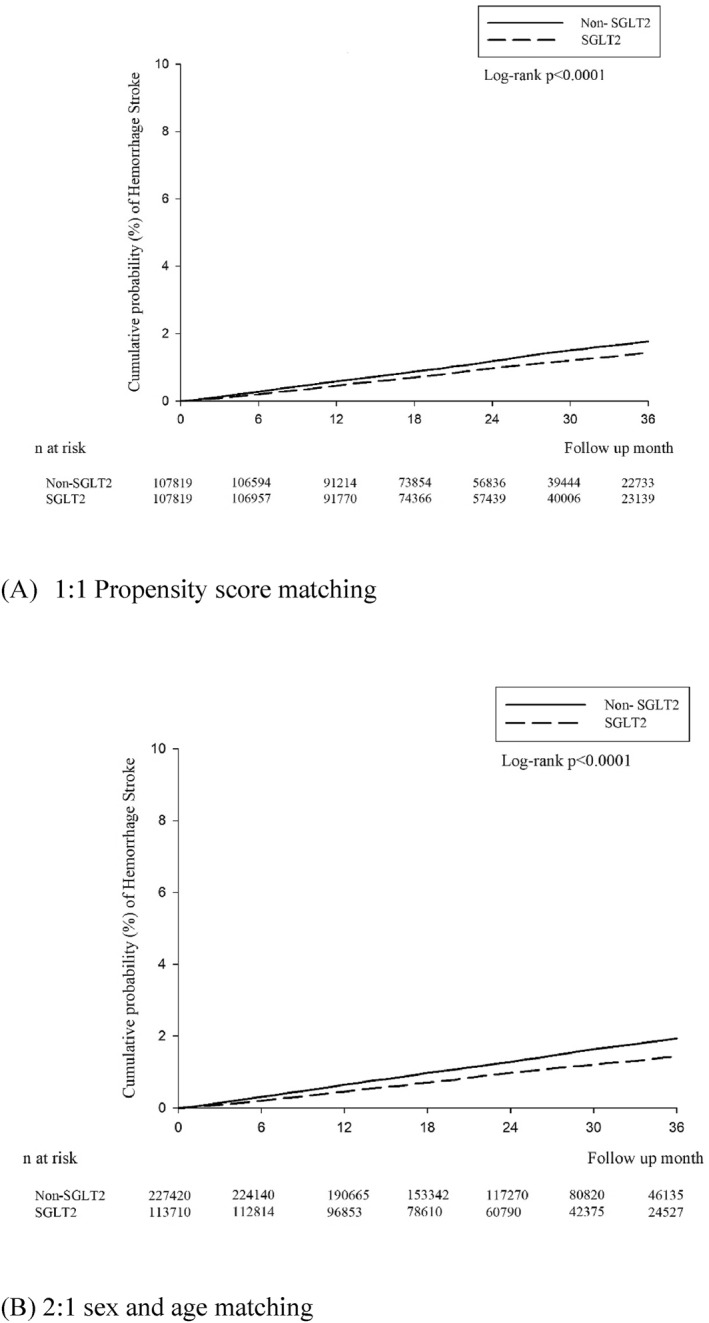
The cumulative incidence of haemorrhagic stroke in a Kaplan–Meier plot.

**FIGURE 3:**
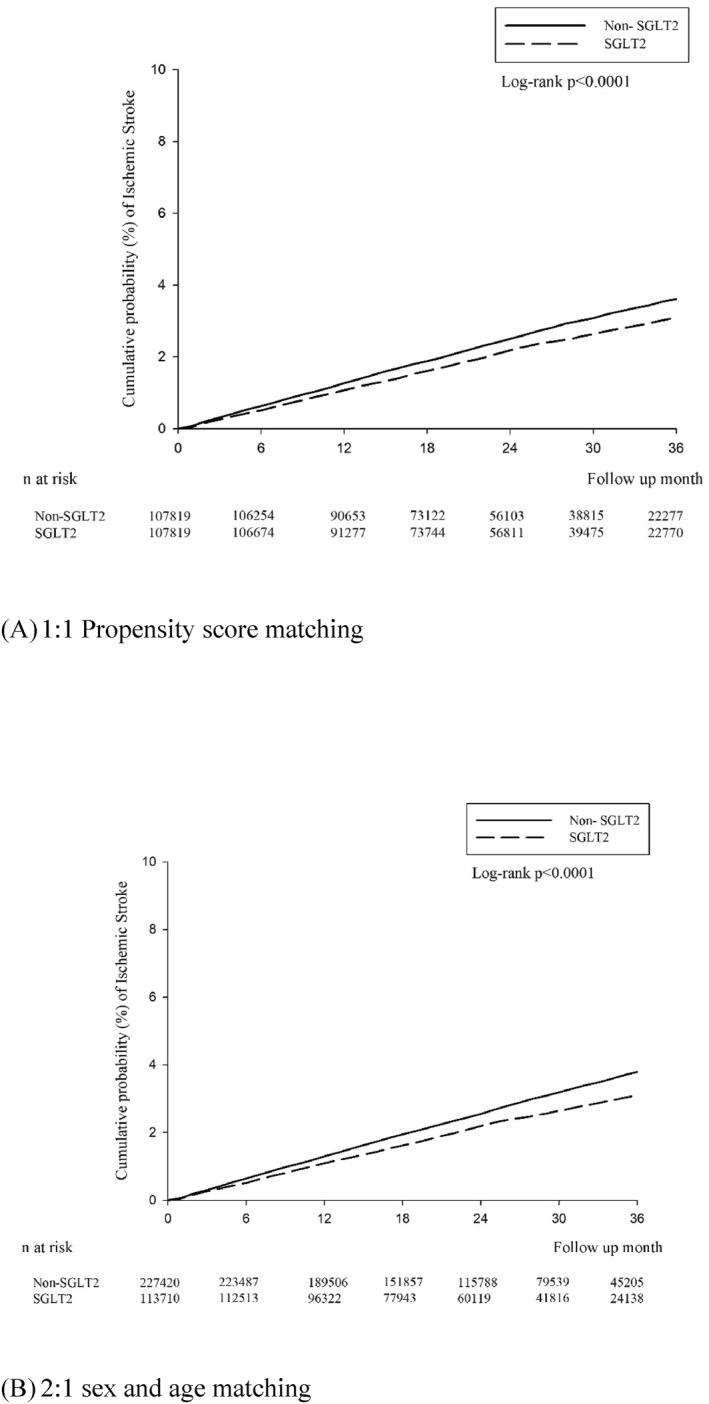
The cumulative incidence of ischaemic stroke in a Kaplan–Meier plot.

The cumulative incidence of ischaemic and haemorrhagic stroke was demonstrated in a Kaplan–Meier plot (Figures 2 and 3). The Kaplan–Meier analysis revealed that the SGLT2i group had a significantly lower cumulative incidence of ischaemic and haemorrhagic stroke (log‐rank, all *p* < 0.0001).

### 3.3 Sensitivity Analysis of the Relative Risk of Ischaemic and Haemorrhagic Stroke in Patients Matched for Sex and Age at a 1:2 Ratio

The median follow‐up period was 2.0 years between the SGLT2i group and the non‐SGLT2i group. The incidence rates of ischaemic and haemorrhagic stroke were 9.30 per 10,000 person‐months (95% CI 8.94–9.67) and 4.18 per 10,000 person‐months (95% CI 3.94–4.43) respectively for SGLT2i users compared with 11.20 (95% CI 10.92–11.49) and 5.63 per 10,000 person‐months (95% CI 5.44–5.84) respectively for non‐SGLT2i users. Simultaneously, there was a significantly lower incidence rate of ischaemic and haemorrhagic stroke in the SGLT2i group compared with the non‐SGLT2i group ([crude HR: 0.83, 95% CI: 0.79–0.87]; [crude HR: 0.74, 95% CI: 0.69–0.80]; Tables 2 and 3). The results did not change after adjustments for sex, age, the index year, comorbidities, and concurrent medication at baseline ([adjusted HR: 0.85; 95% CI: 0.81–0.89]; [adjusted HR: 0.80; 95% CI: 0.74–0.86]).

The Kaplan–Meier analysis consistently revealed that the SGLT2i group had a significantly lower cumulative incidence of ischaemic and haemorrhagic stroke compared with the non‐SGLT2i group (log‐rank, all *p* < 0.0001).

## 4 Discussion

This population‐based cohort study demonstrated that SGLT2i users had a significantly lower risk for ischaemic and haemorrhagic strokes than non‐SGLT2i users among patients with T2D and CKD. Kaplan–Meier and sensitivity analyses also showed a significantly lower risk for ischaemic and haemorrhagic strokes among users than among nonusers of SGLT2i.

SGLT2i therapy is recommended as the preferred glucose‐lowering treatment for reducing cardiovascular and kidney disease risk in patients with T2D, as shown by its benefits for cardiorenal outcomes in cardiovascular outcome trials [16, 17, 18, 19]. However, the effect of SGLT2i on reducing the risk of ischaemic and haemorrhagic strokes in patients with T2D and CKD remains unclear [20, 21, 22]. The previous CREDENCE study [11] and a meta‐analysis of cardiovascular outcome trials of SGLT2i in T2D confirmed that SGLT2i did not significantly reduce ischaemic stroke but showed benefits in preventing haemorrhagic stroke compared with nonusers of SGLT2i [23]. A meta‐analysis of the EMPA‐RAG [24] OUTCOME, CANVAS [25], DECLARE‐TIMI 58 [26], VERTIS CV, [27] and CREDENCE trials demonstrated a significant advantage of active treatment with SGLT2i, which was associated with a significant 51% reduction (relative risk [RR] = 0.49, 95% CI 0.30–0.82, *p* = 0.007). However, it did not demonstrate a significant benefit for ischaemic stroke (HR = 0.87, 95% CI 0.69–1.09, *p* = 0.23) when compared with nonusers [25]. The difference between our study and prior studies, it may be a population‐based, large sample size and using propensity score matching to control for potential confounders in this study. Moreover, no study has reported the association between SGLT2i and the risk of ischaemic or haemorrhagic stroke. To the best of our knowledge, this is the first study to comprehensively examine the risk of ischaemic or haemorrhagic stroke risk among patients with T2D and CKD using SGLT2i.

The mechanisms for the benefits of SGLT2i on ischaemic and haemorrhagic strokes in patients with T2D with CKD still need to be elucidated. SGLT2i may provide cardiorenal benefit through the inhibition of sodium and glucose reabsorption, leading to improvement in both volume and glycaemic control and reduction of intraglomerular hypertension [28]. The primary EMPA‐KIDNEY [29] and EMPA‐KIDNEY follow‐up trials [30] demonstrated that empagliflozin was beneficial in patients regardless of their diabetes status and across estimated glomerular filtration rate categories, with a carryover effect that lowers the risk of progression to end‐stage kidney disease. In vitro data also suggest that SGLT2 improves glucose‐induced vascular dysfunction by reducing oxidative stress and inflammation, reversing pro‐inflammatory phenotypes, modulating mitochondrial function, and glucotoxicity in diabetic rats [31]. However, more studies are needed to understand the protective mechanisms of SGLT2i against ischaemic and haemorrhagic strokes in patients with T2D and CKD.

### 4.1 Limitations

This study has some limitations. First, the National Health Insurance Research Database (NHIRD) does not include laboratory data, such as blood sugar levels, haemoglobin A1c levels, liver function, renal function, and electrocardiogram, and brain computed tomography scans were not available in the claims data. Second, some information was not provided by the NHIRD, such as smoking history, alcohol intake, body mass index, and physical activity. These unmeasured confounders in our data analysis could have influenced the outcome, even after balancing the baseline clinical characteristics by propensity score matching. However, given that population‐based data were used, we assumed the lack of significant difference between users and nonusers of SGLT2i. Third, all participants were residents of Taiwan; therefore, the findings may not apply to patients in other countries. Further prospective randomised controlled trials may be needed in other countries.

## 5 Conclusion

Compared with nonusers of SGLT2i among patients with T2D and CKD, the use of SGLT2i demonstrated a protective effect against ischaemic and haemorrhagic strokes in a real‐world setting. These findings highlight the need for further research regarding the long‐term benefits of SGLT2i in patients with T2D and CKD.

## Author Contributions

Ya‐Hui Lin, Tsung‐Kun Lin, Tsung‐Yuan Yang, and Gwo‐Ping Jong were responsible for the conception and design of the study. Tsung‐Kun Lin and Pei‐Lun Liao conducted the analysis. Ya‐Hui Lin, Tsung‐Kun Lin, and Pei‐Lun Liao were responsible for the interpretation of the data. Ya‐Hui Lin, Tsung‐Yuan Yang, and Gwo‐Ping Jong were responsible for the acquisition of data and supervised the management of data. Tsung‐Kun Lin depicted tables and figures. Ya‐Hui Lin, Tsung‐Yuan Yang, and Gwo‐Ping Jong draughted the manuscript. Tsung‐Yuan Yang, and Gwo‐Ping Jong reviewed and edited the manuscript. All authors critically appraised the manuscript and approved the final version.

## Funding

This study was supported by a grant from Chung Shan Medical University Hospital (CSH‐2020‐C‐001).

## Conflicts of Interest

The authors declare no conflicts of interest.

## Data Availability

All the re‐identified data are available upon reasonable request (cgp8009@yahoo.com.tw).
